# SUV39H1 interacts with HTLV-1 Tax and abrogates Tax transactivation of HTLV-1 LTR

**DOI:** 10.1186/1742-4690-3-5

**Published:** 2006-01-13

**Authors:** Koju Kamoi, Keiyu Yamamoto, Aya Misawa, Ariko Miyake, Takaomi Ishida, Yuetsu Tanaka, Manabu Mochizuki, Toshiki Watanabe

**Affiliations:** 1 Laboratory of Tumor Cell biology, Department of Medical Genome Sciences, Graduate School of Frontier Sciences, The University of Tokyo, 4-6-1 Shirokanedai, Minato-ku, Tokyo 108-8639, Japan; 2 Department of Ophthalmology and Visual Science, Graduate School, Tokyo Medical and Dental University, 1-5-45 Yushima, Bunkyo-ku, Tokyo 113-8519, Japan; 3 Department of Immunology, Graduate School of Medicine, University of the Ryukyus, Okinawa 903-0215, Japan

## Abstract

**Background:**

Tax is the oncoprotein of HTLV-1 which deregulates signal transduction pathways, transcription of genes and cell cycle regulation of host cells. Transacting function of Tax is mainly mediated by its protein-protein interactions with host cellular factors. As to Tax-mediated regulation of gene expression of HTLV-1 and cellular genes, Tax was shown to regulate histone acetylation through its physical interaction with histone acetylases and deacetylases. However, functional interaction of Tax with histone methyltransferases (HMTase) has not been studied. Here we examined the ability of Tax to interact with a histone methyltransferase SUV39H1 that methylates histone H3 lysine 9 (H3K9) and represses transcription of genes, and studied the functional effects of the interaction on HTLV-1 gene expression.

**Results:**

Tax was shown to interact with SUV39H1 *in vitro*, and the interaction is largely dependent on the C-terminal half of SUV39H1 containing the SET domain. Tax does not affect the methyltransferase activity of SUV39H1 but tethers SUV39H1 to a Tax containing complex in the nuclei. In reporter gene assays, co-expression of SUV39H1 represses Tax transactivation of HTLV-1 LTR promoter activity, which was dependent on the methyltransferase activity of SUV39H1. Furthermore, SUV39H1 expression is induced along with Tax in JPX9 cells. Chromatin immunoprecipitation (ChIP) analysis shows localization of SUV39H1 on the LTR after Tax induction, but not in the absence of Tax induction, in JPX9 transformants retaining HTLV-1-Luc plasmid. Immunoblotting shows higher levels of SUV39H1 expression in HTLV-1 transformed and latently infected cell lines.

**Conclusion:**

Our study revealed for the first time the interaction between Tax and SUV39H1 and apparent tethering of SUV39H1 by Tax to the HTLV-1 LTR. It is speculated that Tax-mediated tethering of SUV39H1 to the LTR and induction of the repressive histone modification on the chromatin through H3 K9 methylation may be the basis for the dose-dependent repression of Tax transactivation of LTR by SUV39H1. Tax-induced SUV39H1 expression, Tax-SUV39H1 interaction and tethering to the LTR may provide a support for an idea that the above sequence of events may form a negative feedback loop that self-limits HTLV-1 viral gene expression in infected cells.

## Background

Human T-cell leukemia virus type 1 (HTLV-1) is the causative agent of an aggressive leukemia known as adult T-cell leukemia (ATL), as well as HTLV-1 associated myelopathy/tropical spastic paraparesis (HAM/TSP) and HTLV-1 uveitis (HU). These diseases develop usually after more than 40 years of clinical latency [1-4]. No or little, if any, viral gene expression can be detected in the peripheral blood of HTLV-1 carriers or ATL cells, indicating that HTLV-1 is infected latently *in vivo *[5,6].

The viral protein Tax plays a central role in the development of diseases mentioned above in HTLV-1-infected carriers. Tax can activate transcription of the HTLV-1 genome as well as specific cellular genes including inflammatory cytokines and their receptors and adhesion molecules. Tax also shows transforming activity when expressed in T lymphocytes and fibroblasts [7-10]. Tax is a 40-kDa nuclear phosphoprotein which is translated from a spliced HTLV-1 mRNA transcribed from the 3' portion of the genome. Tax regulates multiple cellular responses by its protein-protein interactions with various host cellular factors. In the regulation of transcription, Tax does not bind DNA directly but stimulates transcription from the HTLV-1 LTR and from the promoters of specific cellular genes by recruiting cellular transcription factors. Tax-mediated transcriptional regulation is based on its interaction with DNA-binding transcription factors such as members of the cyclic AMP response element binding protein/activating transcription factor (CREB/ATF), the nuclear factor-κB (NF-κB), and the serum response factor (SRF) and with two related transcriptional co-activators CREB binding protein (CBP) and p300.

In order to activate transcription of the HTLV-1 genome, nuclear Tax interacts with the CREB/ATF family of transcriptional activators, which bind to the viral long terminal repeat (LTR) [11-14]. The interaction of Tax with CREB and the CREB response elements in the LTR results in a CREB response element-CREB-Tax ternary complex [10]. Tax also binds directly to the KIX domain of the transcriptional co-activators CREB-binding protein (CBP) and p300 [15,16]. CBP and p300 are histone acetylases and acetylate substrates such as histones and transcription factors and may serve as integrators of numerous cellular signaling processes with the basal RNA polymerase II machinery [17,18]. This would, in turn, allow controlled regulation and interaction with many cellular transcription factors including CREB, NF-κB/Rel, p53, c-Myb, c-Jun, c-Fos, and transcription factor IIB in a signal-dependent and, sometimes, mutually exclusive fashion. In this context, Tax-mediated repression of transcription of some cellular genes are explained by functional competition between transcription factors and Tax [19]. A recent report that Tax interacts with a histone deacetylase (HDAC) [20] showed a novel mechanism by which Tax represses transcription of certain target genes. HDAC1 is likely to compete with CBP in binding to Tax and functions as a negative regulator of the transcriptional activation by Tax.

Reversible modification of core histones plays an important role in the regulation of gene expression, such as acetylation, phosphorylation and methylation [21,22]. These covalent modifications, alone or in combination, act as a scaffold for the recruitment of specific regulatory proteins or protein complexes that participate in certain downstream nuclear process including transcription, replication and repair [23]. Thus, it is thought that this "histone code" may serve to establish and maintain distinct chromosomal domains that are epigenetically transmitted [24,25]. Consistent with the histone code, it has been revealed that the methylation of histone H3 lysine 9 (H3 K9), a modification associated with transcriptionally silent heterochromatin, is critical for long-range chromatin regulatory processes [26,27]. Several enzymes are known to methylate H3 K9, such as murine SUV39H1 and G9a proteins [28,29].

Although regulation of histone acetylation by Tax through its physical interaction with histone acetylases and deacetylases has been reported, functional interaction of Tax with histone methyltransferases (HMTase) has not been studied. Here we examined the ability of Tax to interact with a histone methyltransferase SUV39H1 and studied the functional effects of the interaction on HTLV-1 gene expression. We report that Tax interacts with SUV39H1 *in vitro*, and that a stronger binding is observed when mutant proteins retain the C-terminal half of SUV39H1 encompassing the SAC (SET-associated Cys-rich) and SET domains of SUV39H1 [30,31]. Our data indicate that Tax interaction does not affect the methytransferase activity of SUV39H1, but induces a relocalization of SUV39H1 in the nuclei resulting in colocalization with Tax. Furthermore, co-expression of SUV39H1 with N-terminal deletion mutant of Tax resulted in cytoplasmic distribution of both proteins. We further demonstrate that SUV39H1 represses Tax transactivation of HTLV-1 LTR promoter activity depending on the Suv39H1 methyltransferase activity and revealed induction of SUV39H1 expression by Tax and tethering of induced SUV39H1 to the HTLV-1 LTR. These data suggest a possible negative feedback loop of HTLV-1 gene expression in infected cells, which may be one of the bases for the induction of HTLV-1 latency.

## Results

### HTLV-1 Tax interacts with SUV39H1

**Figure 1:**
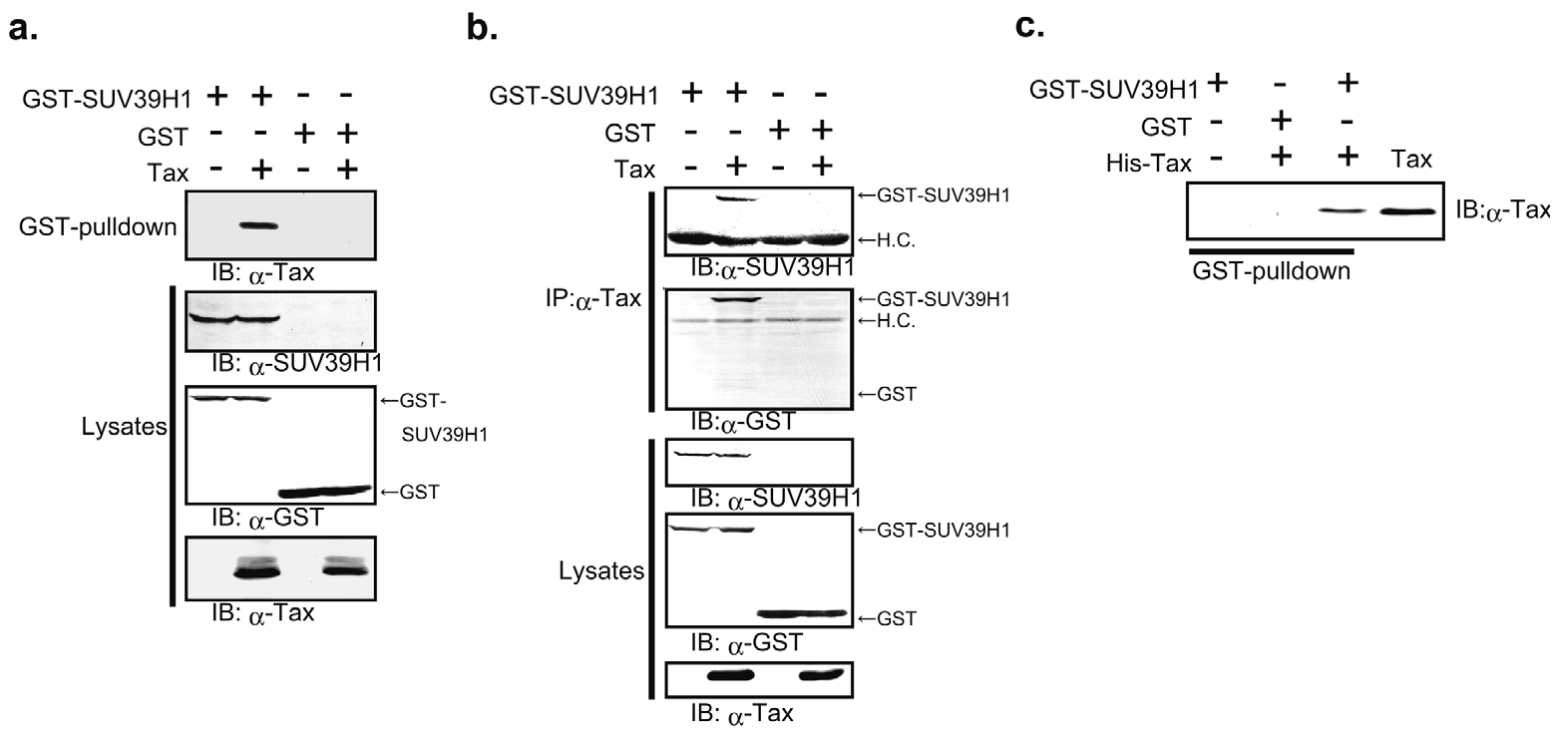
Tax interacts with SUV39H1 in vitro. (a) HEK293T cells were transiently cotransfected with GST-SUV39H1 or GST and Tax. After 48 h, the cells were lysed and the proteins were affinity purified with Glutathione Sepharose 4B. Purified proteins were separated by SDS-PAGE, transferred to a PVDF membrane, and probed with anti-Tax antibody Lt-4 (top panel). Expression of transduced proteins was confirmed by immunoblot analyses of whole cell lysates using respective antibodies (lower panels). (b) HEK293T cells were transiently co-transfected with expression plasmids, GST-SUV39H1 or GST and Tax. After 48 h, the cells were lysed and the proteins were immunoprecipitated with Lt-4. The immunoprecipitates were separated by SDS-PAGE, transferred to a PVDF membrane, and probed with anti-SUV39H1 or anti-GST antibody (upper panels). Expression of proteins was confirmed by immunoblot analyses of whole cell lysates using respective antibodies (lower panels). (c) Direct interaction between SUV39H1 and Tax. Bacterially expressed GST-SUV39H1 and GST were purified with Glutathione Sepharose 4B, and histidine-tagged wild type Tax (His-Tax) was purified with ProBond Resin (Promega). GST-SUV39H1 and GST were bound to Glutathione Sepharose 4B, and mixed with purified His-Tax in PBS. After centrifugation, proteins bound to Glutathione Sepharose 4B were separated by electrophoresis, transferred to a PVDF membrane, and probed with anti-Tax antibody. As a control, an aliquot of purified His-Tax was run in lane 4. IP, immunoprecipitation; IB, immunoblot; H.C., heavy chain

To determine whether HTLV-1 Tax has the ability to interact with SUV39H1, we used GST pull-down and co-immunoprecipitation assays by transient transfection of expression vectors for these proteins. Transient transduction was used for the experiments because the assays were not sufficiently sensitive with endogenous proteins and others also encountered this problem [32]. Expression vectors for the wild type HTLV-1 Tax (pCG-Tax) and GST-tagged SUV39H1 (pMEG-SUV39H1) were transfected into HEK293T cells as described in Materials and Methods. GST-SUV39H1 protein was affinity purified using Glutathione-Sepharose 4B column from total cellular proteins. Co-purified proteins were analyzed by immunoblotting using anti-Tax monoclonal antibody Lt-4 [33]. Total cellular proteins were also analyzed by immunoblotting as controls for protein expression using antibodies for SUV39H1, Tax and GST proteins. The results clearly showed that affinity-purified GST-SUV39H1 complex contained HTLV-1 Tax protein, whereas Tax protein was not co-purified with GST alone (Figure 1a). Conversely, when the cell lysates were immunoprecipitated with anti-Tax antibody Lt-4, the immune complex was shown to contain SUV39H1 that was detected by anti-SUV39H1 antibody as well as anti-GST antibody (Fig. 1b, upper two panels). Absence of Tax protein in the immune complex when GST protein alone was co-expressed denied the possibility that Tax might be co-immunoprecipitated because of the affinity to GST protein (Fig. 1b, lane 4). Taken together, these results suggested that wild type Tax interacts with SUV39H1 in cultured cells.

Next, we examined direct interaction between Tax and SUV39H1 using bacterially expressed and purified proteins. GST pull-down assays of histidine-tagged Tax and GST-fusion SUV39H1 were performed for this analysis. The results clearly showed that Tax protein directly interacts with GST-SUV39H1 but not with GST protein alone (Fig. 1c).

### Binding domain analysis

**Figure 2:**
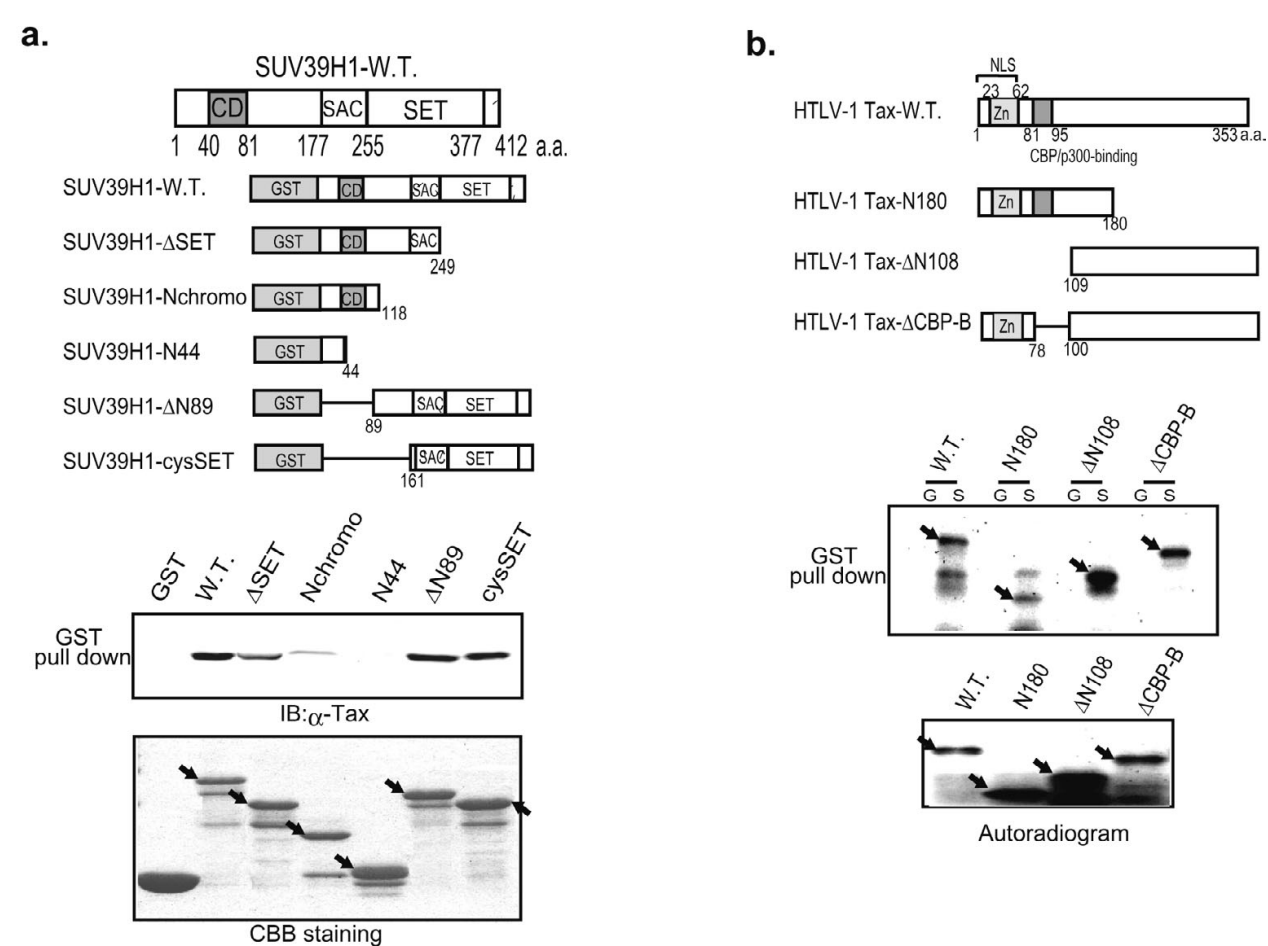
Analyses of the interacting domains. (a) GST pull-down assays using bacterially expressed GST-tagged wild type and various mutants of SUV39H1 and histidine-tagged wild type Tax (His-Tax). A schematic representation of the wild type (SUV39H1-WT) and those of domain structures of mutants are indicated in the upper panels. Results of the pull-down assays are shown in the lower panels. Pulled-down proteins were analyzed by SDS-PAGE and immunoblotting with Lt-4 antibody (top of the lower panels). The bottom panel shows the Coomassie Brilliant Blue (CBB)-stained gel where the wild type and various mutant SUV39H1 proteins were run. (b) Pull-down assays using the wild type GST-SUV39H1 and *in vitro *translated wild type and various mutant Tax proteins. Schematic description of the structures of wild type and various mutant Tax proteins is presented in the top panel. Results of the pull-down assays are shown in the top of the lower panels. Pulled-down Tax proteins that were labeled with ^35^S-methionine were visualized by autoradiography (top of the lower panels). The bottom panel shows the autoradiogram of the gel where the radio labeled wild type and various mutant Tax proteins were run.

To define the domains within SUV39H1 and Tax that are responsible for the interaction, we performed *in vitro *binding assays. First, we constructed various mutants of SUV39H1 according to the domain structure [34] (Fig. 2a, upper panel) and examined binding to the His-tagged wild type Tax protein that was bacterially expressed and purified by ProBond Resin (Promega). When C-terminally deleted series of SUV39H1 were examined, a mutant (ΔSET) that lost the SET domain and the C-terminal cysteine-rich region, but retained the SET-associated Cys-rich (SAC) domain, showed a significantly decreased binding (less than half of the band intensities of the wild type, ΔN108 and ΔCBP-B, when measured by NIH Image software). Further deletion up to amino acid 118 that resulted in loss of the SAC domain (a mutant named Nchromo) showed very weak residual binding activity (about one tenth of the intensities of the wild type, ΔN108 and ΔCBP-B). A mutant retaining only the N-terminal 44 amino acids (N44) totally lost binding activity (Fig. 2a, top of the lower panels, lanes 2 to 5). Two N-terminally deleted mutants (ΔN89 and cycSET) were tested to narrow down the binding region. The ΔN89 mutant lacks the N-terminal region including the chromodomain but retains the region between the chromodomain and the SAC domain (amino acids 89 to 160). The cycSET mutant retains the SAC and SET domains with the C-terminal cysteine-rich region. GST pull-down assays showed that both mutants have strong binding activities, indicating that the loss of the amino acids from 89 to 160 does not affect binding activity. Taken together, although the interaction appears to be complex and may involve several domains, the region of amino acids from 161 to 412 (the SAC-SET domains and C-terminal cysteine-rich region) appears to be enough to show a high affinity for Tax protein. Since the defined region comprises the catalytic motif required for the HMTase activity [34], the results shown above indicate that the catalytic region of SUV39H1 appears to play an important role in the interaction with Tax.

We then analyzed the domains of Tax protein responsible for the interaction with SUV39H1. In addition to the wild type Tax, we used three kinds of mutants, TaxN180, TaxΔN108 and ΔCBP-B. TaxN180 has a C-terminal deletion up to 180 amino acids, TaxΔN108 a deletion of N-terminal 108 amino acids and ΔCBP-B a deletion of the CBP binding domain (amino acids from 79 to 99) (Fig. 2b, upper panel). After *in vitro *translation and labeling with ^35^S-Methionine, the wild type Tax and these mutants were used for *in vitro *pull-down assays with GST-SUV39H1. The results demonstrated that the wild type Tax and all these mutants can bind to SUV39H1 (Fig. 2b, top of the lower panels). However, TaxN180 showed a significantly weaker binding compared with other proteins (about half of the radioactivity of the wild type Tax), suggesting that the C-terminal region of Tax may have a higher affinity for SUV39H1. Furthermore, it was shown that the p300/CBP-binding domain is dispensable for the interaction with SUV39H1 (Fig. 2b, top of the lower panels).

### Co-localization of Tax and SUV39H1 *in vivo*

**Figure 3:**
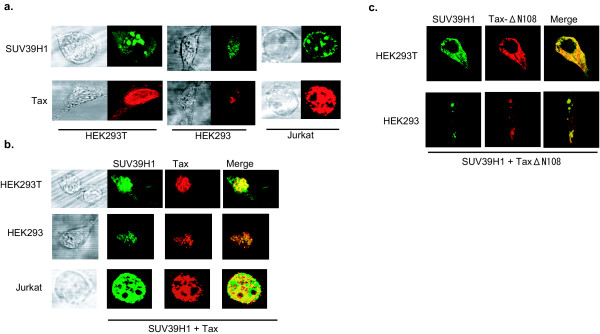
Immunofluorescence microscope analysis of SUV39H1 and Tax. (a) HEK293T, HEK293 and Jurkat cells were cultured on glass coverslips, transfected with SUV39H1 or Tax (upper and lower panels, respectively). Large and defined nuclear speckles were observed in the cells transfected with SUV39H1 (upper panels). Rather diffuse nuclear localization was observed in those transfected with Tax (lower panels). Phase contrast photographs are on the left of each immunofluorescence photograph. (b) HEK293T, HEK293 and Jurkat cells transfected with SUV39H1 and Tax expression plasmids together. Phase contrast photographs are on the left of immunofluorescence photographs. The merged photographs are shown on the right of each panel. (c) HEK293T and HEK293 cells transfected with SUV39H1 and TaxΔN108 together. The merged photograph is shown on the right.

Next, we examined by confocal immunofluorescence analysis whether the intracellular localization of SUV39H1 may be influenced by interaction with Tax. When SUV39H1 alone was transduced in HEK293T, HEK293 and Jurkat cell lines, it showed large and defined nuclear speckles as reported previously [32,35] (Fig. 3a, upper panel). It is known that Tax usually shows speckled nuclear distribution [36,37], whereas in another report it shows diffuse nuclear localization [38]. In our experiments using HEK293T and Jurkat cells, transduced Tax showed diffuse nuclear localization similar to the previous report [38]. (Fig. 3a, lower panel). However, when these two proteins were simultaneously transduced, SUV39H1 protein did not show the speckled distribution and was diffusely distributed within the nuclei and colocalized with transduced Tax in all these cell lines (Fig. 3b). Since the distribution of Tax protein did not appear to have changed in the cells where both proteins were co-expressed, the results suggest a tethering of SUV39H1 by Tax.

To examine the possible tethering of SUV39H1 by Tax, we transduced an N-terminally deleted mutant Tax protein (TaxΔN108) lacking the nuclear localization signal and the wild type SUV39H1 in HEK293T and HEK293 cell lines. Transduced TaxΔN108 showed a clear cytoplasmic distribution as expected (Fig. 3c). In the presence of TaxΔN108, co-expressed SUV39H1 showed a cytoplasmic distribution instead of the nuclear localization seen when expressed alone (Fig. 3c). These results provide supportive evidence for the idea that Tax influences the cellular localization of SUV39H1.

### SUV39H1 methyltransferase activity is not affected by the interaction with Tax

**Figure 4:**
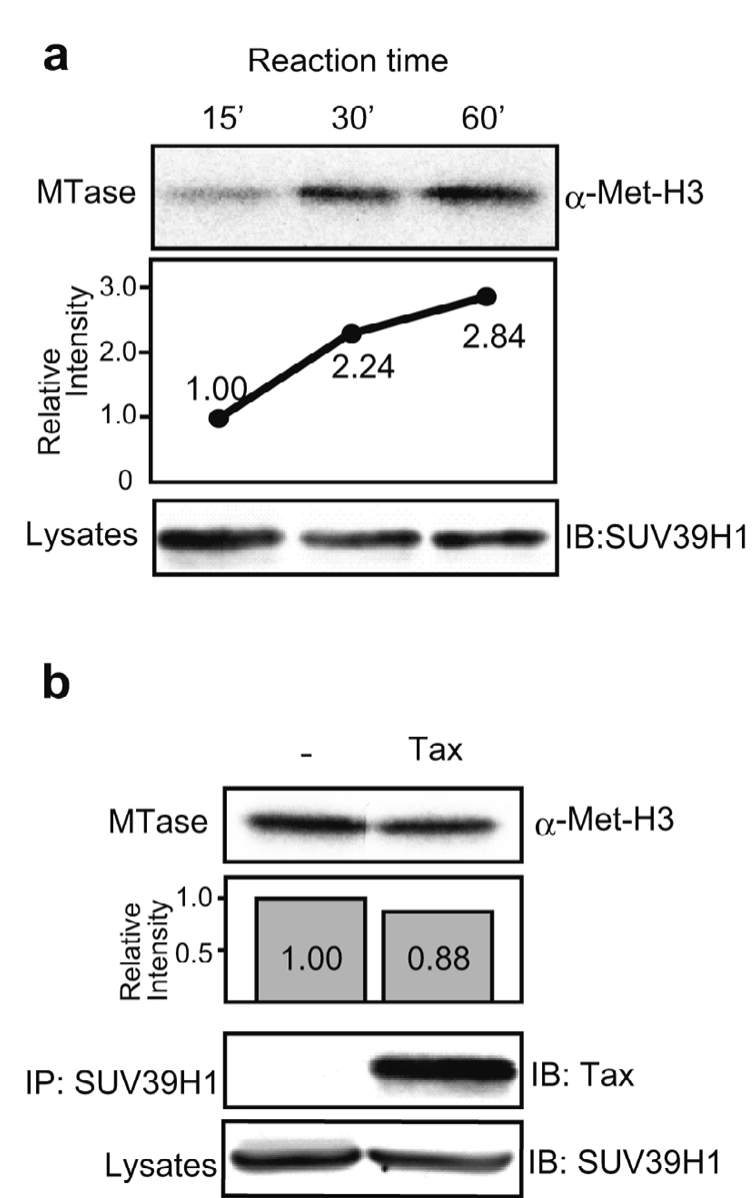
Results of *in vitro *methyltransferase assays. (a) Time course analysis. Top panel shows a representative fluorogram of the reaction mixtures at the indicated time points analyzed by 15% SDS-PAGE. The middle panel shows the relative levels of methylation measured by densitometric analyses of the bands. Bottom panel, a result of immunoblot analysis of transduced SUV39H1 by anti-SUV39H1 monoclonal antibody, showing comparable levels of SUV39H1 expression in each sample. (b) A representative result of three independent experiments of *in vitro *methyltransferase assays of SUV39H1 transduced with or without Tax. The reaction time was 30 min. The second panel shows the relative intensities of the methylated H3 bands. Lower panels show the results of immunoblot analyses of the immunoprecipitates and whole cell lysates to show the presence of SUV39H1 with or without Tax. IP, immunoprecipitation; IB, immunoblot. Antibodies used are indicated on the side of the panels.

When two proteins interact with each other, functional modulation is expected to take place. Thus, we first examined whether association with Tax may affect the HMTase activity of SUV39H1, using *in vitro *methyltransferase assays according to the method reported by Fuks et al. with slight modifications [39]. First, we measured methyltransferase activities of immunoprecipitated SUV39H1 alone that was transduced in HEK293T cells, and studied the time course of the activities (Fig 4a). SUV39H1 immunoprecipitates methylated the substrate H3 (Fig. 4a, top panel). The levels of methylation appeared to become saturated at 60 min and thereafter (Fig. 4a, middle panel). Thus, we performed the reaction for 30 min to examine the effects of Tax on SUV39H1 HMTase activities. When Tax was co-expressed with SUV39H1 in HEK293T cells, the immunoprecipitates showed almost equal levels of methyltransferase activities compared with that of singly expressed SUV39H1 (Fig. 4b, upper two panels). Taken together, these results suggest that although Tax shows a high affinity for the region containing the SET domain of SUV39H1, Tax does not affect the HMTase activity of SUV39H1 under the experimental condition used.

### SUV39H1 represses Tax transactivation of HTLV-1 LTR promoter activity

**Figure 5:**
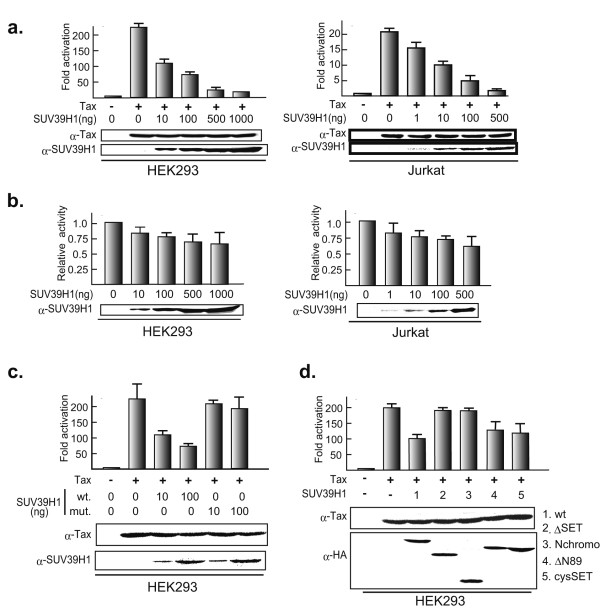
SUV39H1 represses Tax transactivation of HTLV-1 LTR promoter activity. Representative results of luciferase assays using HEK293 and Jurkat cells (left and right panels, respectively) are shown with the mean and standard deviation of triplicate experiments. Below the graphs, results of immunoblot analyses of whole cell lysates are shown to confirm expression of transduced proteins. (a) Dose-dependent repression of Tax transactivation of HTLV-1 LTR by SUV39H1. More than three independent assays were done for each cell line. (b) Effects of SUV39H1 on the basal activities of HTLV-1 LTR. In the absence of Tax, increasing amounts of SUV39H1 expression plasmid was transfected with HTLV-1 Luc. Left and right panels show the results of HEK293 and Jurkat cells, respectively. (c) Absence of repression of Tax transactivation by HMTase negative SUV39H1. Tax expression plasmid was co-transfected with the wild type or HMTase negative mutant SUV39H1 along with the reporter plasmid pHTLV LTR-Luc. Lower two panels show the results of immunoblot analyses to confirm the expression of transduced Tax and SUV39H1 proteins. Antibodies used are indicated on the left. (d) Suppressive activities of SUV39H1 mutants on Tax transactivation of HTLV-1 LTR promoter activity. Fold activation of HTLV LTR promoter activity by Tax is shown with the mean and standard deviation of triplicated experiments. Co-transfected HA-tagged mutant SUV39H1 constructs are indicated below the graph and on the right of lower panels. Structures of these deletion mutants are described in Fig. 2a, upper panel.

Since Tax interacts with and tethers SUV39H1 without affecting HMTase activity, it is possible that SUV39H1 associated with Tax will methylate H3 K9 of the local chromatin where Tax is located, resulting in an interference of Tax function. One of the main biological functions of Tax is transcriptional transactivation of HTLV-1 LTR leading to efficient expression of viral RNA and viral replication in the infected cells. Thus, we examined the effects of SUV39H1 on transactivating function of Tax using pHTLV-LTR-Luc as a reporter. When transduced alone, Tax transactivated the HTLV-1 LTR promoter activity more than 200- and 20-fold in HEK293 and Jurkat cells, respectively. However, when SUV39H1 was co-transduced with Tax, the transactivation was dose-dependently suppressed in both cell lines down to the baseline levels with 500 ng or 1000 ng of the SUV39H1 plasmid (Fig. 5a, left and right panels). On the other hand, SUV39H1 alone showed only a little suppressive activity on the basal activities of HTLV-1 LTR promoter in both cell lines with corresponding amounts of the expression plasmid in the above experiments (Fig. 5b, left and right panels).

Next, we tested whether repression of Tax transactivation by SUV39H1 is dependent on the SUV39H1 methyltransferase activity. For this purpose, we used a loss-of-function mutant of SUV39H1 (H324L) reported by Lachner et al. [40], as well as deletion mutants used for the binding analysis. Co-expression of SUV39H1 (H324L) with Tax did not show a significant suppression of Tax transactivation of HTLV-1 LTR promoter activity (Fig. 5c). Furthermore, co-expression of C-terminal deletion mutants of SUV39H1 (ΔSET, Nchromo and N44) did not show any suppression of Tax transactivation, whereas co-expression of deletion mutants retaining the SAC-SET region (ΔN89 and cysSET) showed suppression of Tax transactivation similar to the levels by the wild type SUV39H1 (Fig. 5c).

Taken together, these results indicate that the interaction between SUV39H1 and Tax leads to repression of Tax transactivating function on HTLV-1 LTR depending on the HMT activity of SUV39H1.

### Induction of SUV39H1 expression by Tax and localization on HTLV-1 LTR

**Figure 6:**
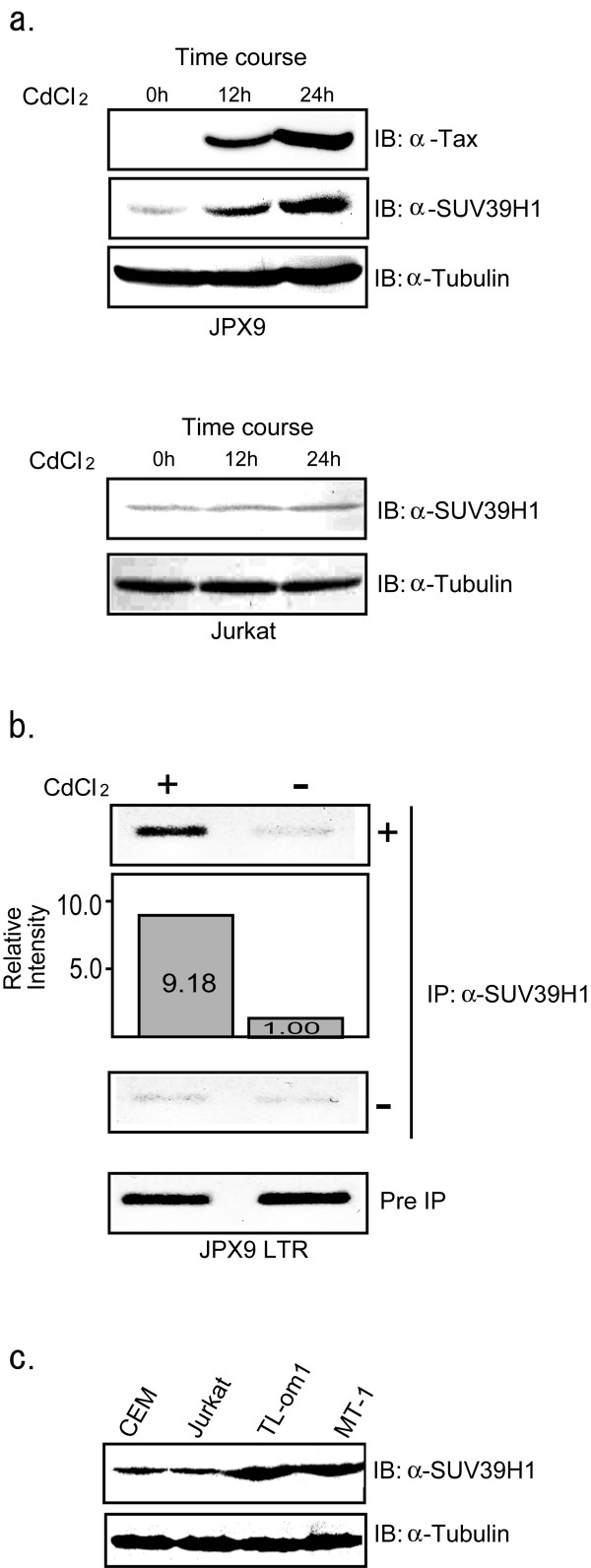
Induction of SUV39H1 expression in JPX9 cells and localization on the HTLV-1 LTR, and endogenous levels of SUV39H1 expression in T cell lines. (a) Top figure: Expression of Tax and SUV39H1 in CdCl_2_-treated JPX9 cells. Whole cell lysates of JPX9 cells treated by CdCl_2 _for indicated periods were studied by immunoblot analysis with anti-Tax and anti-SUV39H1 monoclonal antibodies (top and middle panels). The bottom panel shows the immunoblot by anti-tubulin antibody. Bottom figure: Absence of SUV39H1 induction in Jurkat cells by CdCl_2 _treatment. Whole cell lysates of Jurkat cells treated by CdCl_2 _for indicated periods were studied by immunoblot analysis with anti-SUV39H1 monoclonal antibody (top panel). The bottom panel shows the immunoblot by anti-tubulin antibody. (b) Results of ChIP assays. Representative photographs of agarose gel electrophoresis of PCR products are shown. Top panel shows results of CdCl_2_-treated and untreated JPX9LTR clones. The relative intensities of the band measured by NIH Image software are shown in the second panel. The third and bottom panels show the results of negative controls without first antibody and input controls, respectively. (c) SUV39H1 expression in various T cell lines. ATL-derived cell lines (MT-1 and TL-om1) show higher levels of SUV39H1 expression compared with HTLV-1-uninfected cell lines (top panel). TL-om1 and MT-1 are ATL-derived and HTLV-1-infected cell lines. The bottom panel shows the immunoblot by anti-tubulin antibody.

Above results suggest that SUV39H1 may be a cellular protein counteracting with Tax function. Thus, we next tested the possibility that SUV39H1 expression may be induced by Tax, using JPX9 cells where Tax expression can be induced by CdCl_2 _[41]. As was previously reported, treatment of JPX9 cells with CdCl_2 _resulted in a strong induction of Tax, which was associated with SUV39H1 expression (Fig. 6a, upper figure, upper two panels). Since CdCl_2 _treatment of Jurkat cells, from which JPX9 cells were derived, did not show any effects on the levels of SUV39H1 expression (Fig. 6a, lower figure), SUV39H1 appears to be induced by Tax as one of the Tax target genes.

Next, we examined whether Tax-induction of SUV39H1 leads to localization of SUV39H1 on the HTLV-1 LTR by chromatin immunoprecipitation (ChIP) assays using stable transformants of JPX9 cells transfected with the HTLV-1 LTR Luc plasmid (JPX9LTR clones). PCR analysis showed a clear difference between the ChIP samples of CdCl_2 _treated (48 h) and untreated JPX9LTR clones (Fig. 6b, top panel). The intensity of the band was almost 10-fold stronger in CdCl_2 _treated JPX9LTR cells than that of untreated cells measured by NIH Image software (Fig. 6b, second panel). The intensity of the PCR product from the CdCl_2 _untreated JPX9LTR clones was almost the same as those from the samples of negative control without anti-SUV39H1 antibody (Fig. 6b). These results suggest that, with the induction of Tax expression, at least part of the induced SUV39H1 protein is recruited to the HTLV-1 LTR sequence. Detailed analyses of JPX9LTR clones as to time course of LTR promoter activities, protein expression levels, intracellular localization and so on are now under way in our laboratory, which will be reported in a separate paper.

To examine whether HTLV-1-infected cells express higher levels of SUV39H1, we studied SUV39H1 expression in T cell lines derived from ATL cells (TL-om1 and MT-1) as well as in those without HTLV-1 infection (Jurkat and CEM). The results clearly showed higher levels of SUV39H1 expression in ATL-derived T cell lines compared with T cell lines without HTLV-1 (Fig 6c, upper panel). These results suggest that SUV39H1 is one of the cellular target genes of Tax.

## Discussion

Tax is a multi-functional regulatory protein encoded by HTLV-1. Through a protein-protein interaction, Tax deregulates multiple cellular processes including cell cycle progression, signal transduction and transcriptional regulation, which provide bases for HTLV-1 pathogenicity. In the present study, we demonstrated for the first time the interaction between HTLV-1 Tax and a histone methyltransferase SUV39H1. The interaction was largely dependent on the C-terminal half of the SUV39H1 protein that encompasses the SAC and SET domains and the C-terminal cysteine-rich region. Interaction with Tax did not affect the SUV39H1 HMTase activity in *in vitro *methyltransferase assays. Tax tethered SUV39H1 resulting in colocalization with Tax in the nuclei and in the cytoplasm when an NLS (-) Tax mutant was expressed. These data provide strong supportive evidence for the idea that Tax directs the cellular localization of SUV39H1. Reporter gene assays showed that transduction of SUV39H1 represses Tax transactivation of HTLV-1 LTR promoter activity, which is dependent on the HMTase activity. Furthermore, endogenous SUV39H1 expression appeared to be induced by Tax expression in JPX9 cells, and induced SUV39H1 was shown to be recruited to the HTLV-1 LTR. Taken together, these data may suggest a negative feedback loop of HTLV-1 gene expression in the infected cells, where the transcriptional activator Tax itself may serve as a trigger for a self-limiting control over viral gene expression through the recruitment of SUV39H1 to HTLV-1 LTR and inducing H3 K9 methylation and a repressive histone code on the LTR.

By GST pull-down experiments, the Tax binding domain of SUV39H1 was narrowed down to the region covering the SAC and SET domains (Fig. 2a). On the other hand, the SUV39H1 binding domain of Tax was not clearly defined because all Tax mutants used showed affinities for SUV39H1 (Fig. 2b). However, the results indicated that the N-terminal region of about 100 amino acids of Tax is not essential for a high affinity interaction with SUV39H1 (Fig. 1b). This region contains the nuclear localization signal (NLS) and the CBP binding domain (CBP-B) [38,42]. The CBP-B of Tax does not appear to be involved in the binding to SUV39H1, since the amounts of the pull-down products of the mutants lacking this region (ΔCBP-B and TaxΔN108) were almost equal to that of the wild type (Fig. 2b), and co-expression of SUV39H1 with TaxΔN108 lacking NLS showed cytoplasmic localization of SUV39H1 (Fig. 3c). Many functional domains reside in the region where Tax shows a higher affinity for SUV39H1, such as those involved in the interaction with IKKγ [43], self-dimerization [44], and Rev-like nuclear export signal [45]. Thus, although SUV39H1 shares a functional characteristic with p300/CBP as histone modification enzymes, it appears to interact with Tax in a region distinct from that of p300/CBP. Consequently, the competition model proposed for repression of Tax transactivation by p53 may not be the mechanism by which SUV39H1 represses Tax transactivation.

Tax binding domain of SUV39H1 appears to be located in the C-terminal half region encompassing the SAC-SET and the C-terminal cysteine-rich regions (Fig. 2a). Our results contrast with previous reports showing that the N-terminal region of SUV39H1 is involved in the interaction with other proteins such as HP1b, HPC2, HDAC1 and 2 [31,32,46]. The interaction between SUV39H1 and the above proteins provides a scaffold for a functional multi-protein complex [39,47,48]. Furthermore, the N-terminal domain of 3–118 amino acids is considered the heterochromatin-targeting region. On the other hand, the SET domain is considered a dominant module which regulates SUV39H1 function such as chromatin distribution and protein interaction potentials [31]. The finding that interaction with Tax does not affect HMT activity of SUV39H1 (Fig. 4b) may suggest a new potential to form Tax-containing protein complexes in which above mentioned functions of SUV39H1 are preserved.

It was reported that endogenous SUV39H1 is a heterochromatic protein during interphase that selectively accumulates at centromeric positions of metaphase chromosomes [29,49]. Furthermore, the chromosomal localization of human SUV39H1 is very sensitive to protein expression levels [31]. In the present study, co-expression experiments showed a re-localization of nuclear SUV39H1, losing its typical speckled pattern in the presence of Tax (Fig. 3). SUV39H1 shows a rather diffuse distribution and co-localization with Tax in all cell lines used. These results suggest a possibility that Tax tethers SUV39H1 to the region where Tax is localized (Fig. 3b). This notion is supported by the observation that a mutant Tax lacking the NLS directs cytoplasmic localization of SUV39H1 (Fig. 3c). High levels of expression and coexistence of these proteins can be expected in the cells soon after HTLV-1 infection where the viral gene is vigorously transcribed and abundant Tax protein presumably coexists with high levels of SUV39H1 protein induced by Tax. If Tax tethers SUV39H1, Tax and SUV39H1 may form a repressive complex at the promoter where Tax is localized, thereby SUV39H1 may counteract the transcriptional activation by Tax. Our results of reporter gene assays and ChIP analysis showing dose-dependent repression of Tax transactivation of HTLV-1 LTR and SUV39H1 recruitment to the LTR after Tax induction in JPX9LTR cells provide a supportive evidence for this hypothesis. Thus, a negative feedback loop can be conceived by which HTLV-1 gene expression is made self-limiting. Since SUV39H1 can interact and form a complex with DNA methyltransferases [39], demonstration of SUV39H1 complex on HTLV-1 LTR may also provide a basis for the mechanism of heavy CpG methylation of HTLV-1 LTR in the latently infected cells in the peripheral blood and ATL cells *in vivo *[5].

## Conclusion

In the present paper we demonstrated for the first time the interaction between SUV39H1 and HTLV-1 Tax, and apparent tethering of SUV39H1 by Tax, leading to co-localization in the nuclei. Since Tax interaction does not affect SUV39H1 HMTase activity, Tax-mediated tethering of SUV39H1 to the LTR and induction of a conformational change of the chromatin through H3 K9 methylation can explain the dose-dependent repression of Tax transactivation of LTR by SUV39H1. Taken together with the induction of endogenous SUV39H1 expression by Tax and the recruitment to the LTR, Tax-SUV39H1 interaction may form a negative feedback loop that self-limits HTLV-1 viral gene expression in infected cells

## Materials and methods

### Cell cultures and transfection

Jurkat, HEK293 and HEK293T cell lines were obtained from Fujisaki Cell Biology Center (Okayama, Japan) and the Japanese Cancer Research Resources Bank (Tokyo, Japan). JPX9, a cell line that can be induced to express Tax by CdCl_2 _treatment, was a gift from Prof. Sugamura, Tohoku University. Jurkat and HEK293T cells were cultured in RPMI 1640 supplemented with 10% FCS and antibiotics, and in DMEM supplemented with 10% FCS and antibiotics, respectively. For the co-immunoprecipitation and *in vitro *methyltransferase assays, transfection was done by the standard calcium phosphate precipitation method using 8 × 10^5 ^HEK293T cells and a total of 30 μg of expression vectors. An empty expression vector pME18S or pMEG was used for control transfections or to make the total amount of transfected plasmid to be 30 μg.

### Plasmids and cDNA

Human cDNA for SUV39H1 was amplified by RT-PCR from a normal human PBMC cDNA, and used after confirmation of the nucleotide sequence. The primers used for amplification were as follows: SUV39H1-F1: 5'-CCGCTCGAGATGGCGGAAAATTTAAAAGGCTGCAGCGTG-3', SUV39H1-R1: 5'-GGACTAGTCTAGAAGAGGTATTTGCGGCAGGACTCAGT-3'. GST-fusion proteins of mutants of SUV39H1 that lack functional domains were also prepared using PCR of the wild type cDNA. Forward primers: 5'-AAACTCGAGATGTTCCACAAGGACTTAGAAAGGGAGCTG-3' (ΔN89), 5'-AAACTCGAGATGGTGTACATCAATGAGTACCGTGTTGGT-3' (cysSET), Reverse primers, 5'-GGACTAGTGTCATTGTAGGCAAACTTGTGCAGTGACGC-3' (wild type, ΔN89, cysSET), 5'-CCCACTAGTTCACCGGAAGATGCAGAGGTCATATAGGAT-3' (ΔSET), 5'-CCCACTAGTTCACAGGTAGTTGGCCAAGCTTGGGTCCAG-3' (Nchromo), 5'-CCCACTAGTTCACAGGTAGTTGGCCAAGCTTGGGTCCAG-3' (N44). pGEX5X-3 (Amersham) was used to prepare bacterially expressed GST-fusion proteins. For the expression in mammalian cell lines, the following expression vectors were constructed. pMEG, a vector containing the humanized GST protein [50,51], was used to construct pMEG-SUV39H1, which was used for binding assays. pME-Flag-SUV39H1 was used for transient co-transfection and co-immunoprecipitation assays. For functional and immunohistochemical analyses, an expression vector pcDNA-HA-SUV39H1 was used. To prepare an expression vector for a kinase-negative SUV39H1, we mutated histidine codon 324 into a leucine codon according to Lachner et al [40] using PCR with a mutated primer. The region from nucleotide position 961 from ATG to 1239 (end of the stop codon) was amplified using a mutating forward primer (5'-TTTGTCAACC*T*CAGTTGTGACCCCAACCTGCA-3') and a reverse primer SUV39H1-R1. The amplified fragment replaced the region of the wild type cDNA in pcDNA-HA-SUV39H1 using the HincII restriction enzyme site. The resultant plasmid has a mutated cDNA encoding leucine at 324 instead of histidine (H324L) and was named pcDNA-HA-SUV39H1-H324L. GST-fusion proteins were purified using Glutathione Sepharose 4B (Amersham), followed by confirmation with SDS-PAGE and CBB staining. For expression of histidine-tagged Tax protein, pET3d/Tax was prepared, and the fusion protein was purified by ProBond Resin (Invitrogen), followed by confirmation by SDS-PAGE and CBB staining.

### *in vitro *transcription and translation

For *in vitro *translation of the wild type and mutant Tax proteins, the cDNA was amplified by PCR and cloned into pBluescript II SK (-). *in vitro *transcription and translation of the indicated cDNA was done using TNT QuickCoupled Transcription/Translation Systems (Promega). The proteins were labeled by incorporating ^35^S-Methionine (Amersham), and confirmed by autoradiography of the SDS-PAGE of the products. The primers used are as follows: forward primer for wild type, N180 and ΔCBP-B, 5'-TGAATTCCATATGGCCCACTTCCCAGGGTTTGGA-3', forward primer for ΔN108, 5'-TGAATTCCATATGCGCAAATACTCCCCCTTCCGA-3'; reverse primer for wild type, N180 and ΔCBP-B, 5'-AAACTCGAGGGATCCGACTTCTGTTTCGCGGAAATGTTT-3', reverse primer for N180, 5'-CCCGAGCTGGCCGGGGTCGCAAAA-3'. A Tax mutant that lacks the CBP binding domain (amino acids 81 to 108) was prepared as follows. First, SpeI recognition site was introduced into the nucleotide positions of 238 to 243 and 334 to 339 by Kunkel's method, then the plasmid was digested by SpeI and the larger fragment was separated and recovered from agarose gel, followed by self-ligation. The oligonucleotides used for introduction of point mutations are as follows: MS-1: 5'-CTCCCCTCCTTCCCCACTAGTAGAACCTCTAAGACC-3', MS-2 5'-CAGGCCATGCGCAAAACTAGTCCCTTCCGAAATGGA-3'.

### GST pull-down assay

Wild type and mutant GST-SUV39H1 proteins (2 μg) bound to Glutathione-Sepharose 4B were mixed with His-tagged Tax protein (2 μg) in cold PBS and incubated at 4°C for one hour. After centrifugation, proteins bound to Glutathione-Sepharose 4B were separated by 10% SDS-PAGE followed by immunoblot analysis using anti-Tax monoclonal antibody Lt-4. Relative intensities of the bands were determined using the NIH Image software. Binding analyses using *in vitro *translated and ^35^S-labeled Tax proteins were done basically as described above. The amounts of *in vitro *translation products were one fourth of the reaction mixture. Binding was detected by autoradiography of the dried gel that had been fixed for 30 min in 10% acetic acid, 10% methanol, 10% glycerol followed by treatment with Amplify Fluorographic Reagent (Amersham) for 30 min. Relative intensities of signals were determined by Autoimage Analyzer (BAS2000, Fuji Photo Film, Tokyo).

### Co-immunoprecipitation and immunoblotting

Immunoblots were done to detect co-immunoprecipitated or GST pull-down proteins, as described previously [52]. For co-immunoprecipitation analyses, cell lysates were prepared in TNE buffer (10 mM Tris-HCl, pH7.8, 1% Nonidet P-40, 150 mM NaCl, 1 mM EDTA). When indicated, aliquots were removed for immunoblots of whole cell lysates. Primary antibodies used were anti-SUV39H1 monoclonal antibody (abcam) and anti-Tax monoclonal antibody Lt-4 [33] and alkaline phosphatase-conjugated anti-mouse immunoglobulin sheep and anti-rabbit donkey antibodies (both from Promega) were used as secondary antibodies.

### Immunohistochemistry

HEK293T cells (8 × 10^5^) were grown on coverslips for one day, and transfected with 10 μg of pCG-Tax and 20 μg of pcDNA-HA-SUV39H1 by the calcium phosphate precipitation method. Jurkat cells (2 × 10^5^) were transfected with 2 μg each of pCG-Tax and pcDNA-HA-SUV39H1 plasmids using Lipofectamine2000 (Invitrogen). After 36 hours, HEK293T cells were fixed with 4% paraformaldehyde for 10 min at room temperature, followed by permeabilization with 0.1% TritonX. Jurkat cells were harvested and fixed with acetone/methanol (1:1). Both cells were incubated with anti-Tax antibody Lt-4 and/or anti-HA antibody for one hour, followed by washing with PBS and incubation with fluorescence labeled secondary antibodies for one hour. The secondary antibodies used were Alexa Fluor 546 (anti-mouse antibody, Invitrogen) and Alexa Fluor 488 (anti-rabbit antibody, Invitrogen). Cells were fixed on a slide glass using mounting medium (PBS: glycerol, v:v = 1:9) and covered with a FluoroGuard antifade reagent (Bio-Rad). Fluorescence signals were detected using confocal microscopy (Radiance 2000, Bio-Rad).

### *in vitro *HMT assay

The assay was done basically according to the method reported by Fuks et al. [39] with slight modifications. Briefly, SUV39H1 expression vector pME-Flag-SUV39H1 was transfected alone or with Tax expression vector pCG-Tax into HEK293T cells. After culturing for 40 hours, cells were lysed in TNE buffer, followed by immunoprecipitation with anti-FLAG M2 antibody. The immunoprecipitates were used for *in vitro *methyltransferase assay using histone octamer (Sigma) as substrates. Reaction was done at 30°C for indicated time in a reaction buffer containing 50 mM Tris-HCl, pH8.5, 20 mM KCl, 10 mM MgCl_2_, 10 mM β-mercaptoethanol, and 250 mM sucrose) in the presence of 10 μCi ^3^H-adenosylmethionine (Amersham). The reaction mixture was analyzed by 15% SDS-PAGE. After fixation, gels were treated with Amplify Fluorographic Reagent (Amersham) for 30 min and followed by fluorography. The levels of methylation were evaluated by densitometric analyses of the bands using NIH Image software.

### Reporter Gene Assays

To study the transactivation of HTLV-1 LTR promoter by Tax, reporter gene assays were done, using pHTLV-LTR-Luc plasmid as a reporter and pCG-Tax as an effector in the presence or absence of SUV39H1. pHTLV-LTR-Luc and pCG-Tax were generous gifts from Prof. J Fujisawa, Kansai Medical University [53]. Briefly, a reporter plasmid, pHTLV-LTR-Luc, was constructed by inserting a 647-bp HTLV-1 LTR fragment into the MCS site of the pGL3 vector (Promega). HEK293 cells were transfected with 50 ng of pHTLV-LTR-Luc, 50 ng of pCG-Tax and 10 to 1,000 ng of pcDNA-HA-SUV39H1 by the calcium phosphate precipitation method. Jurkat cells were transfected with 100 ng of pHTLV-LTR-Luc, 10 ng of pCG-Tax and 10 to 500 ng of pcDNA-HA-SUV39H1 by the DEAE method [50]. A β-galactosidase expression plasmid driven by the β-actin promoter (pβ-act-β-gal) [54] was co-transfected to standardize each experiment. Cells were harvested 48 h after transfection, and Luciferase activity was measured with Luciferase assay kit (Promega). The measured activities were standardized by the activities of β-galactosidase, and transactivation was expressed as fold activation compared with the basal activity of LTR-Luc without effectors such as SUV39H1 or Tax. Representative results of triplicate experiments that were repeated more than three times are shown in the figures with the mean and standard deviation.

### Induction of Tax expression in JPX9 cells

JPX9 cells were cultured in RPMI1640 supplemented with 10% FCS and antibiotics unless stimulated with CdCl_2_. Tax expression in JPX9 cells was induced by culturing 1 × 10^6 ^cells in the presence of 30 μM CdCl_2 _for indicated hours. Then, cells were harvested and lysed by 1 × sample buffer (65 mM Tris-HCl pH 6.8, 3% SDS, 10% glycerol, 0.01% BPB) followed by 10 min of boiling. Samples corresponding to 2 × 10^5 ^cells were separated by SDS-PAGE and transferred to PVDF membrane as described above. After blocking with skim milk, the membranes were incubated with the primary antibody at room temperature for one hour, washed in TBST buffer and incubated with the alkaline phosphatase-conjugated anti-mouse secondary antibody at room temperature for one hour. The primary antibodies used are as follows: anti-SUV39H1 mouse monoclonal antibody (abcam), anti-Tax mouse monoclonal antibody Lt-4, and anti-tubulin mouse monoclonal antibody (Santa Cruz).

### Chromatin immunoprecipitation (ChIP) assays

To examine the tethering of SUV39H1 by Tax, we prepared JPX9 transformants that were stably transfected with the HTLV-1 LTR Luc plasmid and pA-puro plasmid. After cloning by limiting dilution, isolated clones were tested for induction of Tax expression and luciferase activities by CdCl_2 _treatment, and selected clones were named JPX9LTR clones (Detailed analyses using these clones will be reported in a separate paper). Using three JPX9 LTR clones, ChIP assays were performed to test tethering of SUV39H1 to the HTLV-1 LTR after Tax expression. Cells (2 × 10^6 ^per clone) were treated with or without CdCl_2 _for 48 hours followed by cross-linking at 37°C for 10 min with formaldehyde (1% final concentration). Cells were pelleted by centrifugation and resuspended in 1 ml of ice-cold PBS (-) with protease inhibitor cocktail (Sigma). Cells were again pelleted by centrifugation at 4°C. The pellet was suspended in 500 μl of the lysis buffer (1% SDS, 10 mM EDTA, 50 mM Tris-HCl [pH8.1]) and kept on ice for 10 min. After sonication on ice with an Astrason Ultrasonic Processor (Misonix) to shear DNA to lengths of between 200 and 1,000 bp (as estimated by agarose gel electrophoresis), lysates were cleared by centrifugation. The supernatant was then diluted 10-fold with dilution buffer (0.01% SDS, 1.1% Triton, 1.2 mM EDTA, 16.7 mM Tris-HCl [pH 8.1], 16.7 mM NaCl) with protease inhibitors to a final volume of 5 ml. An aliquot (500 μl) of the supernatant was saved to represent unfractionated chromatin. The diluted cell supernatant was precleared with a 50% suspension of protein G Sepharose beads (Sigma) for 30 minutes at 4°C with agitation. Sepharose was pelleted by brief centrifugation and the supernatant was transferred to a new tube. The cross-linked chromatin suspension was mixed with anti-SUV39H1 antibody or PBS as negative controls, and incubated overnight at 4°C. Immune complexes were reacted for 1 h at 4°C with agitation with a 50% suspension of protein G-Sepharose beads equilibrated with dilution buffer. After the reaction, the beads were collected and washed serially with the following buffers: buffer a [0.1% SDS, 1% Triton X-100, 2 mM EDTA, 20 mM Tris-HCl[pH8.1], 150 mM NaCl], buffer b [0.1% SDS, 1% Triton X-100, 2 mM EDTA, 20 mM Tris-HCl[pH8.1], 500 mM NaCl], buffer c [0.25 M LiCl, 1% NP-40, 1% Sodium Deoxycholate, 1 mM EDTA, 10 mM Tris-HCl[pH8.1], and TE. Immune complexes were eluted twice with 250 μl of elution buffer (1% SDS, 0.1 M NaHCO_3_) for 15 min at room temperature and 20 μl of 5 M NaCl was added to the 500 μl eluates. Cross-links were reversed by heating at 65°C for 4 h, followed by addition of 10 μl 0.5 M EDTA, 20 μl 1 M Tris-HCl [pH6.5], and incubated at 45°C for 1 h in the presence of 40 μg/ml of proteinase K. The DNA was purified by phenol/chloroform extraction followed by ethanol precipitation. The recovered DNA was resuspended in 50 μl of TE. PCR was performed in 50 μl with Ampli Taq (Perkin-Elmer) and for 35 cycles (annealing temperature is 55°C). The set of primers used was as follows; forward 5'-ACAGAAGTCTGAGAAGGTCA -3' and reverse 5'-TGGGTGGTTCCCGGTGGCTT -3'. The predicted PCR product length is 150 bp. All PCR signals stained with Ethidium Bromide on 2.0% agarose gel were quantified with the NIH Image software.

## Competing interests

The author(s) declare that they have no competing interests.

## Authors' contributions

KK carried out co-immunoprecipitation assays, confocal immunofluorescence analysis and reporter gene assays, and prepared the figures. KY participated in construction of mutant expression plasmids, and performed *in vitro *binding assays. Aya M participated in construction of a methyltransferase negative mutant of SUV39H1 performed a part of reporter gene assays. Ari M prepared JPK9 LTR clones and performed ChIP assays. TI participated in the experimental design and data analysis, and performed *in vitro *HMTase assays. YT provided anti-Tax monoclonal antibody Lt-4 and contributed to experimental design and data analysis. MM participated in the experimental design, data analysis and writing of the manuscript. TW conceived of the study, and participated in its design and coordination, and data analysis, as well as in writing the manuscript. All authors have read and approved the final manuscript.
